# Variations in bariatric surgical care pathways: a national costing study on the variability of services and impact on costs

**DOI:** 10.1186/s40608-018-0223-3

**Published:** 2018-12-26

**Authors:** Eleanor Grieve, Ruth M. Mackenzie, Jane Munro, Joanne O’Donnell, Sally Stewart, Abdulmajid Ali, Duff Bruce, Miranda Trevor, Jennifer Logue

**Affiliations:** 1 Health Economics and Health Technology Assessment, Institute of Health and Wellbeing, University of Glasgow, Glasgow, G12 8QQ UK; 2 Institute of Cardiovascular and Medical Sciences, University of Glasgow, Glasgow, G12 8QQ UK; 3 University Hospital Ayr, NHS Ayrshire and Arran, Ayr, KA6 6DX UK; 4 Institute of Health and Wellbeing, University of Glasgow, Glasgow, G12 8QQ UK; 5 Institute of Cardiovascular and Medical Sciences, University of Glasgow, 126 University Place, Glasgow, G12 8TA UK

**Keywords:** Bariatric surgery, Care pathways, Costing study

## Abstract

**Background:**

We undertook a survey of all bariatric centres in Scotland in order to describe current pre- and post-operative care, to estimate their costs and explore differences in financial impact.

**Methods:**

A questionnaire was distributed to each health centre. Descriptive statistics were used to present average cost per patient along with 95% confidence intervals, and the range of costs.

**Results:**

Results show nearly a five-fold difference in costs per patient for pre-operative services (range £226 - £1071) and more than a three-fold difference for post-operative services (range £259 - £896).

**Conclusions:**

There is a lack of evidence base and a clear requirement for the evaluation of bariatric surgical services to identify the care pathways pre- and post-surgery which lead to largest improvements in health outcomes and remain cost-effective.

**Electronic supplementary material:**

The online version of this article (10.1186/s40608-018-0223-3) contains supplementary material, which is available to authorized users.

## Background

The efficacy of bariatric surgery for large-scale, long-term weight loss is well-established [1–3]. Although bariatric surgery is associated with increased healthcare costs, these are outweighed by the expected health benefits of a reduction in onset of new diabetes, remission of existing diabetes and lower mortality [1–3]. Pre- and post-operative care is a major component of the total cost of bariatric surgery. Anecdote suggests that bariatric surgery care pathways vary considerably, including access to psychology. International bariatric guidance, whilst based on best practice, is not specific on what the optimal model of care is [4] and there is little evidence of whether intensive pre- and post-operative care improves outcomes and is cost-effective compared to less intensive care.

The first steps to obtaining better evidence of what works is to establish what is currently delivered. We undertook a survey of all bariatric centres in Scotland in order to describe current services, to estimate their costs and explore differences in financial impact. This is a necessary first step to facilitate further investigation as to what extent the intensity of pre- and post- operative bariatric surgical care is a factor which may affect patient outcomes after surgery. Scotland continues to have one of the highest prevalence of severe obesity in the world, with 4% of the population having a body mass index (BMI) greater or equal to 40 kg/m^2^, representing an increase in morbid obesity [4] of approximately double from 2008 [5]. This is the first comprehensive study to describe the variation in pre and post-operative bariatric surgical care and the corresponding costs**.**

## Methods

To establish pre-operative assessment and post-operative care pathways used in bariatric surgery services in Scotland, a questionnaire was distributed to each health centre (Additional file 1). This covered pathways for referral, eligibility criteria, the different components of service delivery, the professionals involved, and frequency and length of sessions and consultations. The questionnaire was developed as part of the SurgiCal Obesity Treatment Study (SCOTS) [1] and was presented to all health centres in a group setting at a SCOTS investigator meeting. This ensured each centre understood what information was being sought and why. The questionnaire was then emailed out and responses collected over a 2 year period which served as a consistency check for within centre reporting over multiple years. Follow-up discussions by phone and email were undertaken with centres where clarifications were required, on staffing grade for example. A limitation is that we did not observe practise at any site to cross validate with the self-reported information.

Costs were based on publicly available information for staff time. Unit costs were taken from the Personal Social Services Research Unit (PSSRU) 2015 [6] and the Information and Statistics Division Scotland tariffs 2015 [7]. Cost was calculated per person participating in the bariatric surgery care pathway by multiplying the salary costs of staff, according to grade or band, by the average number of annual sessions provided by that staff member and taking into account the length of session. Multi-Disciplinary Team (MDT) costs were calculated from the number, type and grade of different specialists involved according to their time spent on delivering these sessions. All group sessions were cost per person by taking the average number of patients expected to participate. We make the assumption that costs such as those of equipment and instruments are constant. Rather, the variability in costs is in the staffing and this is what is more likely to affect patient outcomes. Descriptive statistics were used to present average cost per patient along with 95% confidence intervals, as well as the range of costs. Data was costed in Excel and statistical analyses were conducted using Stata version 12. A base case cost was calculated as the most likely average cost per person; a maximum cost was calculated based on optional or additional patient-dependent consultations. We assume zero optional or additional sessions in the base case and at least two for the maximum cost scenario analysis. Where length of sessions or consultations was not provided, 30 min was assumed based on other responses received.

## Results

All ten centres provided information on their bariatric surgery services. The questionnaires were completed, generally by the bariatric dietician or nurse, and returned by email or hard copy to the investigator. Most patients are referred via GPs, diabetes clinics or consultants. Age range was 18–60 years old. Each centre’s bariatric surgery pre- and post-operative care pathways and eligibility criteria regarding glycaemic contol and target weight loss pre-surgery were compared 1 (Table 1). Note that one centre (centre 10) specifies sleep apnoea. This is not costed in our calculations as a cost of surgery but a cost related to an obesity-condition that would have been treated regardless of the bariatric surgery.

**Table 1:** A comparison of Scotland’s tier 4 pathways by bariatric centre

Pre-surgery targets	Centre 1	Centre 2	Centre 3	Centre 4	Centre 5	Centre 6	Centre 7	Centre 8	Centre 9	Centre 10
Pre-surgery targets – weight loss, glycaemic control (targets not specified by NICE). Most boards stated other factors such as attendance rates at clinics and lifestyle changes.	5% weight loss, Hba1c < 64 mmol/mol	5–10% weight loss. Hba1c – control but no target. Smoking cessation. Remittance binge eating.	10% weight loss, Hba1c < 69 mmol/mol	5% weight loss in tier 3, further 5% weight loss tier 4. Hba1c – control but no target.	Weight loss > 5 kg in 6 months	5–10% weight loss. If < 50 BMI, must reach target. Hba1c < 64 mmol/mol	Weight loss > 5 kg, Hba1c < 75 mmol/mol	Weight loss > = 5 kg	Weight loss > 5%, HbA1c < 75 mmol/mol	10% weight loss. Glycaemic control < 75 mmol/mol Hb1c. Smoking cessation. Sleep apnoea 3 months treatment.
Pre-operative assessment ^a^										
Assessment of any psychological or clinical factors that may affect adherence to postoperative care requirements (such as changes to diet).	MDT including clinical psychologist	Dietician, clinical psychologist	Dietician only	Dietician, clinical psychologist	Dietician, clinical psychologist	MDT including clinical psychologist	MDT including clinical psychologist	MDT includes diabetologist	MDT including clinical psychologist	MDT including clinical psychologist
Psychological support	Yes	Yes	No	Yes	If required	Yes	Yes	No	Yes	Yes
Format of sessions (Group or 1:1)	Group	1:1	Either	Both	1:1	Both	Both	1:1	Both	Both
Post-operative assessment:										
Regular postoperative assessment, including specialist dietetic and surgical follow-up	Yes	Yes	Yes	Yes	Yes	Yes	Yes	Yes	Yes	Yes
Psychological support	As required	Not stated	Not stated	Yes	If required	Yes	Yes	No	As required	Not stated
Format of sessions (Group or 1:1)	1:1	1:1	1:1	Both	1:1	1:1	Both	1:1	1:1	1:1

^a^in addition to a risk–benefit analysis by the hospital specialist and/or bariatric surgeon

Results of a sensitivity analysis show nearly a five-fold difference in costs per patient for pre-operative services (range £226 - £1071) and more than a three-fold difference for post-operative services (range £259 - £896) (Table 2). The provision of services was variable regarding the format of delivery of sessions (group or as one-to-one sessions), and frequency and length of access to psychology and dietetics before and after surgery. Access to psychological support was variable both pre- and post-op, with sessions lasting from 30 min to 2 h if this was actually provided. Similarly, for dieticians, some centres offered a one-off appointment pre-op, whilst others provided a regular group service over a number of weeks. Post-op follow-up was more consistent with regular reviews by dieticians, though this was far from standardised across centres. (The full cost breakdown is provided in Additional file 2.)

**Table 2:** Costs of Tier 4 Pathways classified as low medium and high intensity

Health Board	Pre-op (Base Case)	Pre-op (SA)	Post-op (Base Case)	Post-op (SA)	Intensity
1	£681	£1071	£458	£526	High
2	£212	£423	£259	£259	Low
3	£185	£231	£452	£458	Medium
4	£340	£359	£225	£261	Low
5	£138	£226	£414	£483	Medium
6	£408	£798	£209	£356	Medium
7	£498	£544	£339	£339	Medium
8	£472	£472	£339	£339	Medium
9	£425	£539	£248	£896	High
10	£478	£478	£398	£398	Medium
Tier 4 summary costs	Mean	SE	95% CI	Min/Max	
Pre-op (Base Case)	£384	£53	£264, £503	£138, £681	
Pre-op (SA)	£514	£81	£331, £697	£226, £1071	
Post-op (Base Case)	£334	£30	£266, £402	£209, £458	
Post-op (SA)	£432	£59	£299, £564	£259, £896	

Base case = average number of appointments

Sensitivity analysis = maximum number of consultations

Assumed surgical assessment of 20–40 min where not stated by 4 boards

## Discussion

Anecdote suggests that bariatric surgery care pathways vary considerably, including access to psychology. International bariatric guidance, whilst based on best practice, is not specific on what the optimal model of care is [8] . Here we illustrate the large nationwide variability in pre- and post-operative care, a likely consequence of widespread uncertainty regarding best practice and a lack of more detailed guidance regarding service delivery. There is little evidence as to whether intensive pre- and post-operative care improves outcomes and is cost-effective compared to less intensive care. This is likely to be more complicated than one standard pathway for all, with patient preferences also paramount in terms of type of provision (one-to-one or group sessions, for example). Furthermore, pre-surgery targets also vary widely [9] but are often low cost group interventions and funded from a separate budget to surgery. Maximum cost is around £100–£200 per patient. However, these targets do add to the complexity of the pathway for the patients and variation in time and access to surgery. The usefulness of these targets is currently a subject of debate [10–13] and will be investigated as part of SCOTS.

Impacts resulting from the benefits of dietician and psychological support pre- bariatric surgery have been published. Livhits et al. [14], undertook a systematic review which found that pre-operative weight loss would appear to be associated with greater weight loss post-operatively. In a more recent review, Gerber et al. [15] found the same beneficial effects from pre-operative weight loss. On the other hand, it has been shown that psychological support pre- and post- bariatric surgery had no impact on weight loss [16]. This study recommends further research to evaluate the longer-term implications for both weight loss and psychological support, and thereby, the most effective timing for delivery of these interventions. As to why some centres offer more comprehensive services than others, we would suggest that decisions on staff resourcing are being made on the basis of cost and availability of specialists as there is currently no evidence either way as to whether or not these different models of care pathways improve outcomes. Indeed, this study illustrates how variable these costs are, even across health centres within the same country context, and this difference alone is worth highlighting. We, therefore, believe it is important to evidence outcome of these services.

Furthermore, there is a concern that bariatric surgery cost-effectiveness models may either omit pre- and post-surgery care costs as part of their economic analyses or treat patients and the delivery of these services homogenously by applying average costs. In a recent systematic review of a critical appraisal of economic evaluations of bariatric surgery [17], the considerable heterogeneity of what costs are included in economic studies and the frequent omission of different types of healthcare resource use was highlighted. Despite the identification of pre- and post-operative costs, there was no detail reported on care pathways explicitly as an important cost component of an economic evaluation of bariatric surgery. A recent study by Gulliford et al. [2] estimating the costs of bariatric surgery drawn from UK National Health Service (NHS) tariffs, included pre-operative weight management as part of the cost of the surgical procedure but only referred to the cost of medical weight management services. There was no reference to bariatric surgery care pathway costs being included [2]. In the same model, a flat rate of £875 was also included for post-operative reviews. We do not capture the procedure costs and assume this to be relatively standardised given the clear guidance on surgical procedures and, in Scotland, there is national procurement so device costs would also be standard across all sites. In their systematic review, Picot et al., 2009 [3] found the costs of bariatric surgery generally to be presented as standard unit costs with aggregate costs differing dependent on what is included in the total costs of surgery rather than any differences due to centre variation. One study [18] did find variation by gender but offered no explanation as to why.

Our interest is in understanding whether differences in these care pathways are predictors of health outcomes, and thus influence cost-effectiveness from the benefit side. This study underlines the need to better understand the cost-effectiveness of bariatric surgery care pathways, and whether the varying level of intensity of services offered is an important factor in influencing outcomes. As stated above, this work was developed as part of SCOTS, a longitudinal cohort study of bariatric surgery across Scotland. The SCOTS study will allow us the follow-up data required to assess whether this classification of pre- and post-op care pathways is a predictor of health outcomes. Classification of the intensity of pre- and post- operative bariatric surgical care can now be considered for investigation as a factor which may affect patient outcomes after surgery. If further findings do demonstrate that more intensive (and expensive) services lead to better outcomes, we do not envisage this changing bariatric surgery from being cost-effective at the usual willingness-to-pay thresholds for reimbursement on the NHS. However, budgetary impact is an important consideration and we acknowledge that these costs do matter for payers, hospital resource use and more local level decision-making. Should these pathways be found to be predictors of better health outcomes, the case for investment in these care pathways would be self-evident.

## Conclusions

This study, focusing on pre-operative costs and the first 12 months following surgery in which the majority of costs will occur, has illustrated the large nationwide variability in pre- and post-operative care pathways across Scotland, and the subsequent financial impact on the provision of bariatric surgery services. This is a likely consequence of widespread uncertainty regarding best practice and a lack of more detailed guidance regarding service delivery. Health economic analyses do not always capture these costs [17] or apply a flat rate [2]. There is a lack of evidence base and a clear requirement for the evaluation of bariatric surgical services to identify the care pathways pre- and post-surgery which lead to the largest improvements in health outcomes and remain cost-effective to the health provider. Further research to be undertaken as part of the SCOTS study will allow us to investigate this.

## Additional files

Additional file 1: SCOTS Bariatric Surgery Care Pathway Costing Template. This file contains the questionnaire distributed to each health centre in order to establish pre-operative assessment and post-operative care pathways used in bariatric surgery services in Scotland. Questions relate to pathways for referral, eligibility criteria, the different components of service delivery, the professionals involved, and frequency and length of sessions and consultations. (DOC 60 kb)

Additional file 2: Detailed Cost Breakdown by Health Centre (Board). This file provides the full cost breakdown of bariatric surgery care pathways for all ten centres using information from completed questionnaires. (DOC 360 kb)

## Abbreviations

NHS: National Health Service
NICE: National Institute of Health and Care Excellence
PSSRU: Personal Social Services Research Unit
SCOTS: SurgiCal Obesity Treatment Study

## Footnotes

1 We assume that BMI and co-morbidity eligibility criteria would comply with NICE guidance.

## References

[1] Logue J, Stewart S, Munro J, Bruce J, Grieve E, Lean M, Lindsay RS, Bruce D, Ali A, Briggs A (2015). SurgiCal obesity treatment study (SCOTS): protocol for a national prospective cohort study of patients undergoing bariatric surgery in Scotland. BMJ Open.

[2] Gulliford MC, Charlton J, Booth HP, Fildes A, Khan O, Reddy M, Ashworth M, Littlejohns P, Prevost AT, Rudisill C (2016). Health services and delivery research. Costs and outcomes of increasing access to bariatric surgery for obesity: cohort study and cost-effectiveness analysis using electronic health records.

[3] Picot J, Jones J, Colquitt JL, Gospodarevskaya E, Loveman E, Baxter L, Clegg AJ (2009). The clinical effectiveness and cost-effectiveness of bariatric (weight loss) surgery for obesity: a systematic review and economic evaluation. Health Technol Assess.

[4] Global Database on Body Mass Index. https://www.who.int/nutrition/databases/bmi/en/. Accessed 20 Dec 2018.

[5] Bromley C, Corbett J, Day J, Doig M, Gharib W, Given L, Leyland A, MacGregor A, Marryat L, Maw T, et al. In: Bromley C, Given L, editors. Scottish health survey 2010, vol. 1. Edinburgh: A National Statistics Publication for Scotland; 2011.

[6] Curtis L, Burns A (2015). Unit costs of health and social care 2015. Personal Social Services Research Unit.

[7] Scottish National Tariff 2013. Edinburgh: Information Service Division; 2013. https://www.isdscotland.org/Health-Topics/Finance/Publications/2013-10-29/2013-10-29-tariff-summary.pdf. Accessed 20 Dec 2018.

[8] Busetto L, Dicker D, Azran C, Batterham RL, Farpour-Lambert N, Fried M, Hjelmesaeth J, Kinzl J, Leitner DR, Makaronidis JM (2017). Practical recommendations of the obesity management task force of the European Association for the Study of obesity for the post-bariatric surgery medical management. Obes Facts.

[9] Read S, Logue J (2016). Variations in weight management services in Scotland: a national survey of weight management provision. J Public Health.

[10] Schneider A, Hutcheon DA, Hale A, Ewing JA, Miller M, Scott JD (2018). Postoperative outcomes in bariatric surgical patients participating in an insurance-mandated preoperative weight management program. Surg Obes Relat Dis.

[11] Keith C, Goss L, Blackledge C, Stahl R, Grams J (2016). Insurance-mandated pre-operative diet and outcomes following bariatric surgery. Surg Obes Relat Dis.

[12] Houlden RL, Yen JL, Moore S. Effectiveness of an Interprofessional glycemic optimization clinic on preoperative glycated hemoglobin levels for adult patients with type 2 diabetes undergoing bariatric surgery. Can J Diabetes. 2018;42(5):514-19. 10.1016/j.jcjd.2017.12.011. 10.1016/j.jcjd.2017.12.011 29530392

[13] Kalarchian MA, Marcus MD, Courcoulas AP, Cheng Y, Levine MD (2013). Preoperative lifestyle intervention in bariatric surgery: initial results from a randomized, Controlled Trial. Obesity (Silver Spring, Md).

[14] Livhits M, Mercado C, Yermilov I, Parikh JA, Dutson E, Mehran A, Ko CY, Gibbons MM (2009). Does weight loss immediately before bariatric surgery improve outcomes: a systematic review. Surg Obes Relat Dis.

[15] Gerber P, Anderin C, Thorell A (2015). Weight loss prior to bariatric surgery: an updated review of the literature. Scand J Surg.

[16] Ogden J, Hollywood A, Pring C (2015). The impact of psychological support on weight loss post weight loss surgery: a randomised control trial. Obes Surg.

[17] Campbell JA, Venn A, Neil A, Hensher M, Sharman M, Palmer AJ (2016). Diverse approaches to the health economic evaluation of bariatric surgery: a comprehensive systematic review. Obes Rev.

[18] Craig BM, Tseng DS (2002). Cost-effectiveness of gastric bypass for severe obesity. Am J Med.

