# Supplementary material for: Variations in bariatric surgical care pathways: a national costing study on the variability of services and impact on costs
Source: BMC Obes. 2018 Dec 26;5:43. doi: 10.1186/s40608-018-0223-3 (PMC6307243; doi:10.1186/s40608-018-0223-3)
Supplement: Supplementary file 1 — SCOTS Bariatric Surgery Care Pathway Costing Template. This file contains the questionnaire distributed to each health centre in order to establish pre-operative assessment and post-operative care pathways used in bariatric surgery services in Scotland. Questions relate to pathways for referral, eligibility criteria, the different components of service delivery, the professionals involved, and frequency and length of sessions and consultations. (DOC 60 kb) [file 40608_2018_223_MOESM1_ESM.doc]

# Additional File 1

# SCOTS 2015 Bariatric Surgery Care Pathway Update

Completed by:

1. What are current predicted numbers of patients planning bariatric surgery in 2015?
2. What are predicted numbers of patients planning bariatric surgery in 2016?
3. Where do patients from your NHS board have their surgery (if other than home board)? Please specify all possible locations:
4. Please describe who refers patients into the bariatric surgery pathway.
   1. For example do you get direct referrals from GPs, diabetes clinics, or do all patients attend a Tier 3 weight management service before referral. Please include all that apply.
   2. Please briefly describe Tier 3 service if it exists (length, components etc).
5. Describe your current pre-operative care pathway for patients who are planning bariatric surgery. Please describe the particular interventions that patients receive pre-operatively please indicate the clinical team member leading, type of session (one to one, group). Please define the starting point for bariatric surgery:

| Clinical team member | Type of session | Length of session | Frequency/  number of sessions over what period of time | Contents of session  E.g. Training, assessment, behaviour change techniques | If group session, please indicate how many participants on average |
| --- | --- | --- | --- | --- | --- |
|  |  |  |  |  |  |
|  |  |  |  |  |  |
|  |  |  |  |  |  |

Continued from previous page

Post surgery

| Clinical team member | Type of session | Length of session | Frequency/  number of sessions over what period of time | Contents of session  E.g. Training, assessment, behaviour change techniques | If group session, please indicate how many participants on average |
| --- | --- | --- | --- | --- | --- |
|  |  |  |  |  |  |
|  |  |  |  |  |  |
|  |  |  |  |  |  |

1. Please indicate which surgical procedures are undertaken at the site

Gastric band

Gastric bypass

Sleeve gastrectomy

Duodenal Switch

other

1. Do patients have any targets to achieve before they get their surgery (e.g. weight loss or glycaemic control)? When is this target to be achieved (e.g. before referral to Tier 4 or before surgery within Tier 4).
2. What happens if the patients do not achieve these targets?
3. What is planned frequency of band adjustments
4. What is the time post surgery that the band is first filled?

Thank you for taking the time to complete this information.

SCOTS Research Team
