# Supplementary material for: Variations in bariatric surgical care pathways: a national costing study on the variability of services and impact on costs
Source: BMC Obes. 2018 Dec 26;5:43. doi: 10.1186/s40608-018-0223-3 (PMC6307243; doi:10.1186/s40608-018-0223-3)
Supplement: Supplementary file 2 — Detailed Cost Breakdown by Health Centre (Board). This file provides the full cost breakdown of bariatric surgery care pathways for all ten centres using information from completed questionnaires. (DOC 360 kb) [file 40608_2018_223_MOESM2_ESM.doc]

**Additional File 2**

**Full Cost Breakdown**

| **Board 1** | |  | |  | | | |  | | | | | | | |  | | | | | | | |  | |  |  | | | | | |  | | | | | | | | | |  | |  | | | | | | | | | | | |  | |  |
| --- | --- | --- | --- | --- | --- | --- | --- | --- | --- | --- | --- | --- | --- | --- | --- | --- | --- | --- | --- | --- | --- | --- | --- | --- | --- | --- | --- | --- | --- | --- | --- | --- | --- | --- | --- | --- | --- | --- | --- | --- | --- | --- | --- | --- | --- | --- | --- | --- | --- | --- | --- | --- | --- | --- | --- | --- | --- | --- | --- |
| **Clinical team member** | | **Band** | | **Type of session** | | | | **Frequency** | | | | | | | | | | | | | | | |  | | **Length** | **Participants** | | | | | | | | | | | | | | | |  | | **Costs average** | | | | | | | | | | | | **Cost max** | |  |
|  | |  | |  | | | | **Min** | | | | | | | | **Max** | | | | | | | | **Mean** | | **(hours)** | **Min** | | | | | | **Max** | | | | | | | | | | **Mean** | |  | | | | | | | | | | | |  | |  |
| **Pre-op Tier 4** | |  | |  | | | |  | | | | | | | |  | | | | | | | |  | |  |  | | | | | |  | | | | | | | | | |  | |  | | | | | | | | | | | |  | |  |
| Bariatric nurse | | Band 6 | | Outpatient | | | |  | | | | | | | |  | | | | | | | | 1 | | 1.00 |  | | | | | |  | | | | | | | | | | 1 | | £51.00 | | | | | | | | | | | | £51.00 | |  |
| Consultant surgeon | | Consultant | | Clinic | | | | 1 | | | | | | | | 2 | | | | | | | |  | | 0.33 |  | | | | | |  | | | | | | | | | | 1 | | £46.00 | | | | | | | | | | | | £92.00 | |  |
| Clinical psychologist | | Band 8b | |  | | | | 1 | | | | | | | | 2 | | | | | | | |  | | 1.00 |  | | | | | |  | | | | | | | | | | 1 | | £137.00 | | | | | | | | | | | | £276.00 | |  |
| Endoscopy | | Procedure | |  | | | |  | | | | | | | |  | | | | | | | | 1 | | 0.50 |  | | | | | |  | | | | | | | | | |  | | £240.00 | | | | | | | | | | | | £240.00 | |  |
| *MDT* | |  | |  | | | |  | | | | | | | |  | | | | | | | | *6.00* | | *2.00* |  | | | | | |  | | | | | | | | | |  | |  | | | | | | | | | | | |  | |  |
| Dietician | | Band 7 | | Group education | | | |  | | | | | | | |  | | | | | | | | 6 | | 0.29 | 15 | | | | | | 22 | | | | | | | | | |  | | £3.52 | | | | | | | | | | | | £3.52 | |  |
| Nurse | | Band 6 | | Group education | | | |  | | | | | | | |  | | | | | | | | 6 | | 0.29 | 15 | | | | | | 22 | | | | | | | | | |  | | £4.73 | | | | | | | | | | | | £4.73 | |  |
| Psychologist | | Band 8b | | Group education | | | |  | | | | | | | |  | | | | | | | | 6 | | 0.29 | 15 | | | | | | 22 | | | | | | | | | |  | | £12.69 | | | | | | | | | | | | £12.69 | |  |
| Physiotherapist | | Band 6 | | Group education | | | |  | | | | | | | |  | | | | | | | | 6 | | 0.29 | 15 | | | | | | 22 | | | | | | | | | |  | | £3.52 | | | | | | | | | | | | £3.52 | |  |
| Anaesthetist | | Consultant | | Group education | | | |  | | | | | | | |  | | | | | | | | 6 | | 0.29 | 15 | | | | | | 22 | | | | | | | | | |  | | £12.69 | | | | | | | | | | | | £12.69 | |  |
| Endocrinologist | | Consultant | | Group education | | | |  | | | | | | | |  | | | | | | | | 6 | | 0.29 | 15 | | | | | | 22 | | | | | | | | | |  | | £12.69 | | | | | | | | | | | | £12.69 | |  |
| Dietetic Assistant Practitioner | | Band 4 | | Group education | | | |  | | | | | | | |  | | | | | | | | 6 | | 0.29 | 15 | | | | | | 22 | | | | | | | | | |  | | £2.22 | | | | | | | | | | | | £2.22 | |  |
| *MDT* | |  | |  | | | |  | | | | | | | |  | | | | | | | | *1* | | *2.00* |  | | | | | |  | | | | | | | | | |  | |  | | | | | | | | | | | |  | |  |
| Bariatric nurse | | Band 6 | | Outpatient | | | |  | | | | | | | |  | | | | | | | | 1 | | 0.33 |  | | | | | |  | | | | | | | | | | 1 | | £17.00 | | | | | | | | | | | | £17.00 | |  |
| Nurse | | Band 6 | | Outpatient | | | |  | | | | | | | |  | | | | | | | | 1 | | 0.33 |  | | | | | |  | | | | | | | | | | 1 | | £17.00 | | | | | | | | | | | | £17.00 | |  |
| Anaesthetist | | Consultant | | Outpatient | | | |  | | | | | | | |  | | | | | | | | 1 | | 0.33 |  | | | | | |  | | | | | | | | | | 1 | | £45.67 | | | | | | | | | | | | £45.67 | |  |
| Dietician | | Band 7 | | Outpatient | | | |  | | | | | | | |  | | | | | | | | 1 | | 0.33 |  | | | | | |  | | | | | | | | | | 1 | | £12.67 | | | | | | | | | | | | £12.67 | |  |
| Pharmacist | | Band 7 | | Outpatient | | | |  | | | | | | | |  | | | | | | | | 1 | | 0.33 |  | | | | | |  | | | | | | | | | | 1 | | £16.67 | | | | | | | | | | | | £16.67 | |  |
| Cardiographers | | Band 4 | | Outpatient | | | |  | | | | | | | |  | | | | | | | | 1 | | 0.33 |  | | | | | |  | | | | | | | | | | 1 | | £45.67 | | | | | | | | | | | | £45.67 | |  |
| *Other specialists* | |  | |  | | | |  | | | | | | | |  | | | | | | | |  | |  |  | | | | | |  | | | | | | | | | |  | |  | | | | | | | | | | | |  | |  |
| Endocrinologist | | Consultant | | Outpatient | | | | 0 | | | | | | | | 1 | | | | | | | |  | | 0.50 |  | | | | | |  | | | | | | | | | | 1 | |  | | | | | | | | | | | | £68.50 | |  |
| Cardiologist | | Consultant | | Outpatient | | | | 0 | | | | | | | | 1 | | | | | | | |  | | 0.50 |  | | | | | |  | | | | | | | | | | 1 | |  | | | | | | | | | | | | £68.50 | |  |
| Respiratory | | Consultant | | Outpatient | | | | 0 | | | | | | | | 1 | | | | | | | |  | | 0.50 |  | | | | | |  | | | | | | | | | | 1 | |  | | | | | | | | | | | | £68.50 | |  |
| **Post-op Tier 4** | |  | |  | | | |  | | | | | | | |  | | | | | | | |  | |  |  | | | | | |  | | | | | | | | | |  | |  | | | | | | | | | | | |  | |  |
| Bariatric dietician | | Band 7 | | Phone | | | |  | | | | | | | |  | | | | | | | | 2 | | 0.17 |  | | | | | |  | | | | | | | | | |  | | £12.67 | | | | | | | | | | | | £12.67 | |  |
| Bariatric nurse | | Band 6 | | Phone | | | | 1 | | | | | | | | 2 | | | | | | | |  | | 0.17 |  | | | | | |  | | | | | | | | | |  | | £17.00 | | | | | | | | | | | | £17.00 | |  |
| Consultant surgeon | | Consultant | | Clinic | | | |  | | | | | | | |  | | | | | | | | 3 | | 0.50 |  | | | | | |  | | | | | | | | | |  | | £207.00 | | | | | | | | | | | | £207.00 | |  |
| Bariatric nurse | | Band 6 | | Clinic | | | |  | | | | | | | |  | | | | | | | | 3 | | 0.50 |  | | | | | |  | | | | | | | | | |  | | £76.50 | | | | | | | | | | | | £76.50 | |  |
| Bariatric dietician | | Band 7 | | Clinic | | | |  | | | | | | | |  | | | | | | | | 4 | | 0.50 |  | | | | | |  | | | | | | | | | |  | | £76.00 | | | | | | | | | | | | £76.00 | |  |
| Consultant endocrinologist | | Consultant | | Clinic | | | |  | | | | | | | |  | | | | | | | | 1 | | 0.50 |  | | | | | |  | | | | | | | | | |  | | £68.50 | | | | | | | | | | | | £68.50 | |  |
| Psychologist | | Band 8b | | Outpatient | | | | 0 | | | | | | | | 2 | | | | | | | | 1 | | 0.50 |  | | | | | |  | | | | | | | | | |  | |  | | | | | | | | | | | | £68.50 | |  |
| **Board 2** |  | | |  | | | |  | | | | | | | | | | | | | | | | | |  |  | | | | | | | | | | | | | | | | | |  | | | | | | | | | |  | | | |  |
| **Clinical team member** | **Band** | | | **Type of session** | | | | **Frequency** | | | | | | | | | | | | | | | | | | **Length** | **Participants** | | | | | | | | | | | | | | | | | | **Costs average** | | | | | | | | | | **Cost max** | | | |  |
|  |  | | |  | | | | **Min** | | | | | | | **Max** | | | | | | | | **Mean** | | | **(hours)** | **Min** | | | | **Max** | | | | | | | | | **Mean** | | | | |  | | | | | | | | | |  | | | |  |
| **Pre-op Tier 4** |  | | |  | | | |  | | | | | | |  | | | | | | | |  | | |  |  | | | |  | | | | | | | | |  | | | | |  | | | | | | | | | |  | | | |  |
| Dietician | Band 7 | | |  | | | | 1 | | | | | | | 2 | | | | | | | |  | | | 0.75 |  | | | |  | | | | | | | | | 1 | | | | | 28.5 | | | | | | | | | | 57 | | | |  |
| Psychologist | Band 8b | | |  | | | | 1 | | | | | | | 2 | | | | | | | |  | | | 1.00 |  | | | |  | | | | | | | | | 1 | | | | | 137 | | | | | | | | | | 274 | | | |  |
| **Post-op Tier 4** |  | | |  | | | |  | | | | | | |  | | | | | | | |  | | |  |  | | | |  | | | | | | | | |  | | | | |  | | | | | | | | | |  | | | |  |
| Dietician | Band 7 | | | Assessment | | | |  | | | | | | |  | | | | | | | | 6 | | | 0.75 |  | | | |  | | | | | | | | | 1 | | | | | 171 | | | | | | | | | | 171 | | | |  |
| Surgeon | Consultant | | |  | | | |  | | | | | | |  | | | | | | | | 2 | | | 0.25 |  | | | |  | | | | | | | | | 1 | | | | | 69 | | | | | | | | | | 69 | | | |  |
| Dietician/AHP | Band 7 | | |  | | | |  | | | | | | |  | | | | | | | | 2 | | | 0.25 |  | | | |  | | | | | | | | | 1 | | | | | 19 | | | | | | | | | | 19 | | | |  |
| **Board 3** |  | | |  | | | |  | | | | | | | | | | | | | | | | | |  |  | | | | | | | | | | | | | | | | | |  | | | | | | | | | | | |  | |  |
| **Clinical team member** | **Band** | | | **Type of session** | | | | **Frequency** | | | | | | | | | | | | | | | | | | **Length** | **Participants** | | | | | | | | | | | | | | | | | | **Costs average** | | | | | | | | | | | | **Cost max** | |  |
|  |  | | |  | | | | **Min** | | | | | **Max** | | | | | | | | | **Mean** | | | | **(hours)** | **Min** | | | | | **Max** | | | | | | | **Mean** | | | | | |  | | | | | | | | | | | |  | |  |
| **Pre-op Tier 4** |  | | |  | | | |  | | | | |  | | | | | | | | |  | | | |  |  | | | | |  | | | | | | |  | | | | | |  | | | | | | | | | | | |  | |  |
| Dietician | Band 7 | | | Outpatient | | | |  | | | | |  | | | | | | | | | 1 | | | | 1.00 |  | | | | |  | | | | | | | 1 | | | | | | £38.00 | | | | | | | | | | | | £38.00 | |  |
| Dietician | Band 7 | | | Group education | | | |  | | | | |  | | | | | | | | | 8 | | | | 1.00 | 1 | | | | | 5 | | | | | | |  | | | | | | £101.33 | | | | | | | | | | | | £101.33 | |  |
| **Post-op Tier 4** |  | | |  | | | |  | | | | |  | | | | | | | | |  | | | |  |  | | | | |  | | | | | | |  | | | | | |  | | | | | | | | | | | |  | |  |
| Dietician | Band 7 | | | Outpatient | | | |  | | | | |  | | | | | | | | | 2 | | | | 0.50 |  | | | | |  | | | | | | | 1 | | | | | | £38.00 | | | | | | | | | | | | £38.00 | |  |
| Surgeon | Consultant | | | Clinic | | | |  | | | | |  | | | | | | | | | 6 | | | | 0.50 |  | | | | |  | | | | | | | 1 | | | | | | £414.00 | | | | | | | | | | | | £414.00 | |  |
| Health assistant | Band 4 | | | Clinic | | | | 0 | | | | | 1 | | | | | | | | |  | | | | 0.25 |  | | | | |  | | | | | | | 1 | | | | | | £0.00 | | | | | | | | | | | | £6.00 | |  |
| **Board 4** |  | | |  | | | |  | | | | | | | | | | | | | | | | | |  |  | | | | | | | | | | | | | | | | | |  | | | | | | | | | | |  | | |  |
| **Clinical team member** | **Band** | | | **Type of session** | | | | **Frequency** | | | | | | | | | | | | | | | | | | **Length** | **Participants** | | | | | | | | | | | | | | | | | | **Costs average** | | | | | | | | | | | **Cost max** | | |  |
|  |  | | |  | | | | **Min** | | | | **Max** | | | | | | | | | **Mean** | | | | | **(hours)** | **Min** | | | | | | | **Max** | | | | | | | | **Mean** | | |  | | | | | | | | | | |  | | |  |
| **Pre-op Tier 4** |  | | |  | | | |  | | | |  | | | | | | | | |  | | | | |  |  | | | | | | |  | | | | | | | |  | | |  | | | | | | | | | | |  | | |  |
| Surgeon | Consultant | | | Group | | | |  | | | |  | | | | | | | | | 1 | | | | | 2.00 |  | | | | | | |  | | | | | | | | 10 | | | £27.60 | | | | | | | | | | | £27.60 | | |  |
| Dietician | Band 7 | | | Group | | | |  | | | |  | | | | | | | | | 8 | | | | | 1.25 |  | | | | | | |  | | | | | | | | 10 | | | £38.00 | | | | | | | | | | | £38.00 | | |  |
| Psychologist | Band 7 | | | Group | | | |  | | | |  | | | | | | | | | 8 | | | | | 1.25 |  | | | | | | |  | | | | | | | | 10 | | | £137.00 | | | | | | | | | | | £137.00 | | |  |
| Dietician | Band 7 | | |  | | | | 0 | | | | 1 | | | | | | | | |  | | | | | 0.50 |  | | | | | | |  | | | | | | | | 1 | | |  | | | | | | | | | | | £19.00 | | |  |
| Clinical Psychologist | Band 8a | | |  | | | | 1 | | | |  | | | | | | | | |  | | | | | 1.00 |  | | | | | | |  | | | | | | | | 1 | | | £137.00 | | | | | | | | | | | £137.00 | | |  |
| **Post-op Tier 4** |  | | |  | | | |  | | | |  | | | | | | | | |  | | | | |  |  | | | | | | |  | | | | | | | |  | | |  | | | | | | | | | | |  | | |  |
| Dietician | Band 7 | | | phone | | | | 0 | | | | 2 | | | | | | | | |  | | | | | 0.17 |  | | | | | | |  | | | | | | | | 1 | | |  | | | | | | | | | | | £12.67 | | |  |
| Psychologist | Band 7 | | | phone | | | | 0 | | | | 1 | | | | | | | | |  | | | | | 0.17 |  | | | | | | |  | | | | | | | | 1 | | |  | | | | | | | | | | | £22.83 | | |  |
| Dietician | Band 7 | | |  | | | | 1 | | | | 8 | | | | | | | | |  | | | | | 0.50 |  | | | | | | |  | | | | | | | | 1 | | | £152.00 | | | | | | | | | | | £152.00 | | |  |
| Surgeon | Consultant | | |  | | | |  | | | |  | | | | | | | | | 1 | | | | | 0.33 |  | | | | | | |  | | | | | | | | 1 | | | £46.00 | | | | | | | | | | | £46.00 | | |  |
| Psychologist | Band 7 | | | Group | | | |  | | | |  | | | | | | | | | 1 | | | | | 2.00 | 5 | | | | | | | 15 | | | | | | | |  | | | £27.40 | | | | | | | | | | | £27.40 | | |  |
| **Board 5** |  | | |  | | | |  | | | |  | | | | | | | | |  | | | | |  |  | | | | | | |  | | | | | | | |  | | |  | | | | | | | | | | |  | | |  |
| **Clinical team member** | **Band** | | | **Type of session** | | | | **Frequency** | | | | | | | | | | | | | | | | | | **Length** | **Participants** | | | | | | | | | | | | | | | | | | **Costs average** | | | | | | | | | **Cost max** | | | | |  |
|  |  | | |  | | | | **Min** | | | **Max** | | | | | | | | **Mean** | | | | | | | **(hours)** | **Min** | | | **Max** | | | | | | | **Mean** | | | | | | | |  | | | | | | | | |  | | | | |  |
| **Pre-op Tier 4** |  | | |  | | | |  | | |  | | | | | | | |  | | | | | | |  |  | | |  | | | | | | |  | | | | | | | |  | | | | | | | | |  | | | | |  |
| Surgeon | Consultant | | |  | | | |  | | |  | | | | | | | | 2 | | | | | | | 0.50 |  | | |  | | | | | | | 1 | | | | | | | | £138.00 | | | | | | | | | £138.00 | | | | |  |
| Dietician | Band 7 | | |  | | | | 0 | | | 1 | | | | | | | |  | | | | | | | 0.50 |  | | |  | | | | | | | 1 | | | | | | | |  | | | | | | | | | | | | £19.00 | |  |
| Psychologist | Band 8a | | |  | | | | 0 | | | 1 | | | | | | | |  | | | | | | | 0.50 |  | | |  | | | | | | | 1 | | | | | | | |  | | | | | | | | | £68.50 | | | | |  |
| **Post-op Tier 4** |  | | |  | | | |  | | |  | | | | | | | |  | | | | | | |  |  | | |  | | | | | | |  | | | | | | | |  | | | | | | | | |  | | | | |  |
| Surgeon | Consultant | | |  | | | |  | | |  | | | | | | | | 6 | | | | | | | 0.50 |  | | |  | | | | | | | 1 | | | | | | | | £414.00 | | | | | | | | | £414.00 | | | | |  |
| Dietician | Band 7 | | |  | | | |  | | |  | | | | | | | |  | | | | | | |  |  | | |  | | | | | | | 1 | | | | | | | |  | | | | | | | | |  | | | | |  |
| Psychologist | Band 8a | | |  | | | | 0 | | | 1 | | | | | | | |  | | | | | | | 0.50 |  | | |  | | | | | | | 1 | | | | | | | |  | | | | | | | | | £68.50 | | | | |  |
| **Board 6** |  | | |  | |  | | | | | | | | | | | | | | | | | | | |  |  | | | | | | | | | | | | | | | | | |  | | | | | | | |  | | | | | |  |
| **Clinical team member** | **Band** | | | **Type of session** | | **Frequency** | | | | | | | | | | | | | | | | | | | | **Length** | **Participants** | | | | | | | | | | | | | | | | | | **Costs average** | | | | | | | | **Cost max** | | | | | |  |
|  |  | | |  | | **Min** | | | **Max** | | | | | | | | | **Mean** | | | | | | | | **(hours)** | **Min** | | **Max** | | | | **Mean** | | | | | | | | | | | |  | | | | | | | |  | | | | | |  |
| **Pre-op Tier 4** |  | | |  | |  | | |  | | | | | | | | |  | | | | | | | |  |  | |  | | | |  | | | | | | | | | | | |  | | | | | | | |  | | | | | |  |
| Dietician | Band 7 | | | Group | |  | | |  | | | | | | | | | 1 | | | | | | | | 1.50 | 4 | | 5 | | | |  | | | | | | | | | | | | £11.40 | | | | | | | | £11.40 | | | | | |  |
| Surgeon | Consultant | | | Pre-assessment | |  | | |  | | | | | | | | | 1 | | | | | | | | 0.50 |  | |  | | | | 1 | | | | | | | | | | | | £69.00 | | | | | | | | £69.00 | | | | | |  |
| Psychologist | Band 8b | | | Assessment | | 1 | | | 2 | | | | | | | | |  | | | | | | | | 1.50 |  | |  | | | | 1 | | | | | | | | | | | | £205.50 | | | | | | | | £411.00 | | | | | |  |
| Dietician | Band 7 | | | Assessment | |  | | |  | | | | | | | | | 1 | | | | | | | | 1.00 |  | |  | | | | 1 | | | | | | | | | | | | £38.00 | | | | | | | | £38.00 | | | | | |  |
| Dietician | Band 7 | | | Review | | 1 | | | 2 | | | | | | | | |  | | | | | | | | 0.50 |  | |  | | | | 1 | | | | | | | | | | | | £19.00 | | | | | | | | £38.00 | | | | | |  |
| Dietician | Band 7 | | | Group | |  | | |  | | | | | | | | | 4 | | | | | | | | 1.50 | 4 | | 6 | | | |  | | | | | | | | | | | | £45.60 | | | | | | | | £45.60 | | | | | |  |
| Dietician | Band 7 | | | Review | |  | | |  | | | | | | | | | 1 | | | | | | | | 0.50 |  | |  | | | | 1 | | | | | | | | | | | | £19.00 | | | | | | | | £19.00 | | | | | |  |
| Psychologist | Band 8b | | |  | | 0 | | | 1 | | | | | | | | |  | | | | | | | | 0.50 |  | |  | | | | 1 | | | | | | | | | | | |  | | | | | | | | £68.50 | | | | | |  |
| Dietician | Band 7 | | |  | | 0 | | | 1 | | | | | | | | |  | | | | | | | | 0.50 |  | |  | | | | 1 | | | | | | | | | | | |  | | | | | | | | £19.00 | | | | | |  |
| Dietician | Band 7 | | | Group | | 0 | | | 3 | | | | | | | | |  | | | | | | | | 0.75 | 4 | | 6 | | | |  | | | | | | | | | | | |  | | | | | | | | £17.10 | | | | | |  |
| Psychologist | Band 8b | | | Group | | 0 | | | 3 | | | | | | | | |  | | | | | | | | 0.75 | 4 | | 6 | | | |  | | | | | | | | | | | |  | | | | | | | | £61.65 | | | | | |  |
| **Post-op Tier 4** |  | | |  | |  | | |  | | | | | | | | |  | | | | | | | |  |  | |  | | | |  | | | | | | | | | | | |  | | | | | | | |  | | | | | |  |
| Dietician | Band 7 | | |  | |  | | |  | | | | | | | | | 8 | | | | | | | | 0.50 |  | |  | | | | 1 | | | | | | | | | | | | £152.00 | | | | | | | | £152.00 | | | | | |  |
| Psychologist | Band 8b | | |  | | 0 | | | 1 | | | | | | | | |  | | | | | | | | 0.50 |  | |  | | | | 1 | | | | | | | | | | | |  | | | | | | | | £68.50 | | | | | |  |
| Dietician | Band 7 | | | Group | | 0 | | | 3 | | | | | | | | |  | | | | | | | | 0.75 | 4 | | 6 | | | |  | | | | | | | | | | | |  | | | | | | | | £17.10 | | | | | |  |
| Psychologist | Band 8b | | | Group | | 0 | | | 3 | | | | | | | | |  | | | | | | | | 0.75 | 4 | | 6 | | | |  | | | | | | | | | | | |  | | | | | | | | £61.65 | | | | | |  |
| Dietician | Band 7 | | |  | |  | | |  | | | | | | | | | 3 | | | | | | | | 0.50 |  | |  | | | |  | | | | | | | | | | | | £57.00 | | | | | | | | £57.00 | | | | | |  |
| **Board 7** |  | | | |  | |  | | | | | | | | | | | | | | | | | |  | |  | | | | | | | | | | | | | | | | | | |  | | |  | | | | | | | | |  | |
| **Clinical team member** | **Band** | | | | **Type of session** | | **Frequency** | | | | | | | | | | | | | | | | | | **Length** | | **Participants** | | | | | | | | | | | | | | | | | | | **Costs average** | | | **Cost max** | | | | | | | | |  | |
|  |  | | | |  | | **Min** | | | **Max** | | | | | | | **Mean** | | | | | | | | **(hours)** | | **Min** | **Max** | | | | | **Mean** | | | | | | | | | | | | | |  | | |  | | | | | | | | | |
| **Pre-op Tier 4** |  | | | |  | |  | | |  | | | | | | |  | | | | | | | |  | |  |  | | | | |  | | | | | | | | | | | | | |  | | |  | | | | | | | | | |
| Specialist nurse | Band 6 | | | |  | |  | | |  | | | | | | | 6 | | | | | | | | 1.00 | |  |  | | | | | 3 | | | | | | | | | | | | | | £102.00 | | | £102.00 | | | | | | | | | |
| Dietician | Band 6 | | | |  | |  | | |  | | | | | | | 6 | | | | | | | | 1.00 | |  |  | | | | | 3 | | | | | | | | | | | | | | £76.00 | | | £76.00 | | | | | | | | | |
| Clinical psychologist | Band 8b | | | |  | |  | | |  | | | | | | | 1 | | | | | | | | 2.00 | |  |  | | | | | 1 | | | | | | | | | | | | | | £274.00 | | | £274.00 | | | | | | | | | |
| **Post-op Tier 4** |  | | | |  | |  | | |  | | | | | | |  | | | | | | | |  | |  |  | | | | |  | | | | | | | | | | | | | |  | | |  | | | | | | | | | |
| Specialist nurse | Band 6 | | | |  | |  | | |  | | | | | | | 3 | | | | | | | | 0.50 | |  |  | | | | | 1 | | | | | | | | | | | | | | £76.50 | | | £76.50 | | | | | | | | | |
| Dietician | Band 6 | | | |  | |  | | |  | | | | | | | 3 | | | | | | | | 0.50 | |  |  | | | | | 1 | | | | | | | | | | | | | | £57.00 | | | £57.00 | | | | | | | | | |
| Clinical psychologist | Band 8b | | | |  | |  | | |  | | | | | | | 3 | | | | | | | | 0.50 | |  |  | | | | | 1 | | | | | | | | | | | | | | £205.50 | | | £205.50 | | | | | | | | | |
| **Board 8** |  | |  | | | |  | | | | | | | | | |  | | | | | | | |  | |  | | | | | | | | |  | | | | | | | | | |  | |  | | | | | | | | | |  | |
| **Clinical team member** | **Band** | | **Type of session** | | | | **Frequency** | | | | | | | | | |  | | | | | | | | **Length** | | **Participants** | | | | | | | | |  | | | | | | | | | | **Costs average** | | | | | **Cost max** | | | | | | |  | |
|  |  | |  | | | | **Min** | | | | | | | **Max** | | | | | | **Mean** | | | | | **(hours)** | | **Min** | | | | | | | | | | | **Max** | | | | | | **Mean** | |  | | | | | |  | | | | | |  | |
| **Pre-op Tier 4** |  | |  | | | |  | | | | | | |  | | | | | |  | | | | |  | |  | | | | | | | | | | |  | | | | | |  | |  | | | | | |  | | | | | |  | |
| Surgeon | Consultant | | Assessment | | | |  | | | | | | |  | | | | | | 1 | | | | | 0.17 | |  | | | | | | | | | | |  | | | | | | 1 | | £23.00 | | | | | | £23.00 | | | | | |  | |
| Diabetologist | Consultant | | Assessment | | | |  | | | | | | |  | | | | | |  | | | | | 0.33 | |  | | | | | | | | | | |  | | | | | | 1 | | £45.67 | | | | | | £45.67 | | | | | |  | |
| Anaesthetist | Consultant | | Assessment | | | |  | | | | | | |  | | | | | | 1 | | | | | 1.00 | |  | | | | | | | | | | |  | | | | | | 1 | | £137.00 | | | | | | £137.00 | | | | | |  | |
| Dietician | Band 6 | | Assessment | | | |  | | | | | | |  | | | | | | 1 | | | | | 1.00 | |  | | | | | | | | | | |  | | | | | | 1 | | £38.00 | | | | | | £38.00 | | | | | |  | |
| Dietician | Band 6 | | Follow-ups | | | |  | | | | | | |  | | | | | | 12 | | | | | 0.50 | |  | | | | | | | | | | |  | | | | | | 1 | | £228.00 | | | | | | £228.00 | | | | | |  | |
| **Post-op Tier 4** |  | |  | | | |  | | | | | | |  | | | | | |  | | | | |  | |  | | | | | | | | | | |  | | | | | |  | |  | | | | | |  | | | | | |  | |
| Bandfill nurse |  | |  | | | |  | | | | | | |  | | | | | | 9 | | | | | 0.25 | |  | | | | | | | | | | |  | | | | | | 1 | | £236.25 | | | | | | £236.25 | | | | | |  | |
| Dietician |  | |  | | | |  | | | | | | |  | | | | | | 9 | | | | | 0.30 | |  | | | | | | | | | | |  | | | | | | 1 | | £102.60 | | | | | | £102.60 | | | | | |  | |
| **Board 9** |  | |  | | | |  | | | | | | | | | | | | | | | | | |  | |  | | | | | | | | | | | | | | | | | | |  | | | | | |  | | | | | |  | |
| **Clinical team member** | **Band** | | **Type of session** | | | | **Frequency** | | | | | | | | | | | | | | | | | | **Length** | | **Participants** | | | | | | | | | | | | | | | | | | | **Costs average** | | | | | | **Cost max** | | | | | |  | |
|  |  | |  | | | | **Min** | | | | | | | **Max** | | | | | | **Mean** | | | | | **(hours)** | | **Min** | | | | | | | | **Max** | | | | | | **Mean** | | | | |  | | | | | |  | | | | | |  | |
| **Pre-op Tier 4** |  | |  | | | |  | | | | | | |  | | | | | |  | | | | |  | |  | | | | | | | |  | | | | | |  | | | | |  | | | | | |  | | | | | |  | |
| Nurse | Band 6 | | Group | | | |  | | | | | | |  | | | | | | 1 | | | | | 1.00 | | 14 | | | | | | | | 25 | | | | | | 15 | | | | | £3.40 | | | | | | £3.40 | | | | | |  | |
| AHP dietician | Band 6 | | Group | | | |  | | | | | | |  | | | | | | 1 | | | | | 1.00 | | 14 | | | | | | | | 25 | | | | | | 15 | | | | | £2.53 | | | | | | £2.53 | | | | | |  | |
| Psychologist | Band 8c | | Assessment | | | |  | | | | | | |  | | | | | | 1 | | | | | 0.75 | |  | | | | | | | |  | | | | | | 1 | | | | | £102.75 | | | | | | £102.75 | | | | | |  | |
| Surgeon | Consultant | | Assessment | | | |  | | | | | | |  | | | | | | 1 | | | | | 0.75 | |  | | | | | | | |  | | | | | | 1 | | | | | £103.50 | | | | | | £103.50 | | | | | |  | |
| Anaesthetist | Consultant | | Assessment | | | |  | | | | | | |  | | | | | | 1 | | | | | 0.75 | |  | | | | | | | |  | | | | | | 1 | | | | | £102.75 | | | | | | £102.75 | | | | | |  | |
| Dietician | Band 7 | | Assessment | | | |  | | | | | | |  | | | | | | 1 | | | | | 0.75 | |  | | | | | | | |  | | | | | | 1 | | | | | £28.50 | | | | | | £28.50 | | | | | |  | |
| Nurse | Band 6 | | Assessment | | | |  | | | | | | |  | | | | | | 1 | | | | | 0.75 | |  | | | | | | | |  | | | | | | 1 | | | | | £38.25 | | | | | | £38.25 | | | | | |  | |
| *MDT* |  | |  | | | |  | | | | | | |  | | | | | | 1 | | | | | *12.00* | |  | | | | | | | |  | | | | | |  | | | | |  | | | | | |  | | | | | |  | |
| Dietician | Band 7 | | Group | | | |  | | | | | | |  | | | | | | 1 | | | | | 7.00 | | 10 | | | | | | | | 16 | | | | | | 15 | | | | | £17.73 | | | | | | £17.73 | | | | | |  | |
| Nurse | Band 6 | | Group | | | |  | | | | | | |  | | | | | | 1 | | | | | 2.00 | | 10 | | | | | | | | 16 | | | | | | 15 | | | | | £6.80 | | | | | | £6.80 | | | | | |  | |
| Surgeon | Consultant | | Group | | | |  | | | | | | |  | | | | | | 1 | | | | | 1.00 | | 10 | | | | | | | | 16 | | | | | | 15 | | | | | £9.20 | | | | | | £9.20 | | | | | |  | |
| Clinical Psychologist | Band 8c | | Group | | | |  | | | | | | |  | | | | | | 1 | | | | | 1.00 | | 10 | | | | | | | | 16 | | | | | | 15 | | | | | £9.13 | | | | | | £9.13 | | | | | |  | |
| Patient rep | per diems? | | Group | | | |  | | | | | | |  | | | | | | 1 | | | | | 1.00 | | 10 | | | | | | | | 16 | | | | | | 15 | | | | |  | | | | | |  | | | | | |  | |
| Dietician | Band 7 | |  | | | | 0 | | | | | | | 3 | | | | | | 1 | | | | | 1.00 | |  | | | | | | | |  | | | | | | 1 | | | | |  | | | | | | £114.00 | | | | | |  | |
| **Post-op Tier 4** |  | |  | | | |  | | | | | | |  | | | | | |  | | | | |  | |  | | | | | | | |  | | | | | |  | | | | |  | | | | | |  | | | | | |  | |
| Nurse | Band 6/7 | | Phone call | | | |  | | | | | | |  | | | | | | 3 | | | | | 0.50 | |  | | | | | | | |  | | | | | |  | | | | | £76.50 | | | | | | £76.50 | | | | | |  | |
| Dietician/Nurse | Band 6/7 | | Review | | | |  | | | | | | |  | | | | | | 6 | | | | | 0.75 | |  | | | | | | | |  | | | | | | 1 | | | | | £171.00 | | | | | | £171.00 | | | | | |  | |
| Psychologist | Band 8c | |  | | | | 0 | | | | | | | 1 | | | | | |  | | | | | 0.75 | |  | | | | | | | |  | | | | | | 1 | | | | |  | | | | | | £102.75 | | | | | |  | |
| *Ongoing* |  | |  | | | |  | | | | | | |  | | | | | |  | | | | |  | |  | | | | | | | |  | | | | | |  | | | | |  | | | | | |  | | | | | |  | |
| Surgeon | Consultant | |  | | | | 0 | | | | | | | 2 | | | | | |  | | | | | 0.75 | |  | | | | | | | |  | | | | | | 1 | | | | |  | | | | | | £207.00 | | | | | |  | |
| Psychologist | Band 8c | |  | | | | 0 | | | | | | | 2 | | | | | |  | | | | | 0.75 | |  | | | | | | | |  | | | | | | 1 | | | | |  | | | | | | £205.50 | | | | | |  | |
| Nurse | Band 6 | |  | | | | 0 | | | | | | | 2 | | | | | |  | | | | | 0.75 | |  | | | | | | | |  | | | | | | 1 | | | | |  | | | | | | £76.50 | | | | | |  | |
| Dietician | Band 7 | |  | | | | 0 | | | | | | | 2 | | | | | |  | | | | | 0.75 | |  | | | | | | | |  | | | | | | 1 | | | | |  | | | | | | £57.00 | | | | | |  | |
| **Board 10** |  | |  | | | |  | | | | | | | | | | | | | | | | | |  | |  | | | | | | | | | | | | | | | | | | |  | | | | | |  | | | | | |  | |
| **Clinical team member** | **Band** | | **Type of session** | | | | **Frequency** | | | | | | | | | | | | | | | | | | **Length** | | **Participants** | | | | | | | | | | | | | | | | | | | **Costs average** | | | | | | **Cost max** | | | | | |  | |
|  |  | |  | | | | **Min** | | | | | | | **Max** | | | | | | **Mean** | | | | | **(hours)** | | **Min** | | | | | | | | **Max** | | | | | | **Mean** | | | | |  | | | | | |  | | | | | |  | |
| **Pre-op Tier 4** |  | |  | | | |  | | | | | | |  | | | | | |  | | | | |  | |  | | | | | | | |  | | | | | | 1:1 | | | | | £68.50  £69.00  £68.50  £19.00  £25.50  £38.00  £51.00  £138.00 | | | | | | | | | | | |  | |
| | Psychologist | | --- | | Surgeon | | Anesthetist | | Dietician | | Nurse | | *MDT* | | Dietician | | Nurse | | Surgeon | | Band 8c  Consultant  Consultant  Band 7  Band 6  Band 7  Band 6  Consultant | | Assessment  Assessment  Assessment  Assessment  Assessment  Weekly clinic  Weekly clinic  Weekly clinic | | | |  | | | | | | |  | | | | | | 1  1  1  1  1  1  1  1 | | | | | 0.5  0.5  0.5  0.5  0.5  0.5  0.5  0.5 | |  | | | | | | | |  | | | | | | 1  1 | | | | |  | |
|  |  | |  | | | |  | | | | | | |  | | | | | |  | | | | |  | |  | | | | | | | |  | | | | | | 1 | | | | |  | | | | | |  | | | | | |  | |
| **Post-op Tier 4** |  | |  | | | |  | | | | | | |  | | | | | |  | | | | |  | |  | | | | | | | |  | | | | | |  | | | | |  | | | | | |  | | | | | |  | |
| Dietician Nurse | Band 6/7 | | Review | | | |  | | | | | | |  | | | | | | 9 | | | | | 0.5 | |  | | | | | | | |  | | | | | | 1 | | | | | £171 | | | | | |  | | | | | |  | |
| Surgeon | Consultant | | Review | | | |  | | | | | | |  | | | | | | 2 | | | | | 0.5 | |  | | | | | | | |  | | | | | | 1 | | | | | £138 | | | | | |  | | | | | |  | |
| Nurse | Band 6 | | Review | | | |  | | | | | | |  | | | | | | 2 | | | | | 0.5 | |  | | | | | | | |  | | | | | | 1 | | | | | £51 | | | | | |  | | | | | |  | |
| Dietician | Band 7 | | Review | | | |  | | | | | | |  | | | | | | 2 | | | | | 0.5 | |  | | | | | | | |  | | | | | | 1 | | | | | £38 | | | | | |  | | | | | |  | |
